# Exploring extracellular matrix and prostaglandin pathway alterations across varying resection margin distances of right-sided colonic adenocarcinoma

**DOI:** 10.1186/s12885-023-11595-7

**Published:** 2023-12-07

**Authors:** Tharathorn Suwatthanarak, Pariyada Tanjak, Thanawat Suwatthanarak, Onchira Acharayothin, Kullanist Thanormjit, Amphun Chaiboonchoe, Thikhamporn Tawantanakorn, Chainarong Phalanusitthepha, Atthaphorn Trakarnsanga, Asada Methasate, Manop Pithukpakorn, Ryuichi Okamoto, Vitoon Chinswangwatanakul

**Affiliations:** 1 Graduate School of Medical and Dental Sciences, Joint Degree Doctoral Program in Medical Sciences between Tokyo Medical and Dental University, Tokyo, Japan; 2 Mahidol University, Bangkok, Thailand; 3 Division of General Surgery, Department of Surgery, Faculty of Medicine Siriraj Hospital, Mahidol University, 12th Floor, Syamindra Building, 2, Prannok Road, Bangkok Noi, Bangkok, 10700 Thailand; 4 Siriraj Cancer Center, Faculty of Medicine Siriraj Hospital, Mahidol University, Bangkok, Thailand; 5 Siriraj Center of Research Excellence for Systems Pharmacology, Faculty of Medicine Siriraj Hospital, Mahidol University, Bangkok, Thailand; 6 Division of Medical Genetics, Department of Medicine, Faculty of Medicine Siriraj Hospital, Mahidol University, Bangkok, Thailand; 7 Siriraj Genomics, Faculty of Medicine Siriraj Hospital, Mahidol University, Bangkok, Thailand; 8 Department of Gastroenterology and Hepatology, Tokyo Medical and Dental University, Tokyo, Japan

**Keywords:** Different gene expression, Non-tumor tissues adjacent to the tumor, Surgical resection margin, Right-sided colonic adenocarcinoma, RNA analysis

## Abstract

**Background:**

Surgical resection followed by indicated adjuvant therapy offers potential curative treatment in colonic adenocarcinoma. Beyond the well-established seed and soil theory of colon cancer progression, the 'normal-appearing' tissues near the tumor are not genuinely normal and remain as remnants in patients following surgery. Our objective was to elucidate the alteration of gene expression and pathways across various distances of resection margins in right-sided colonic adenocarcinoma.

**Methods:**

Twenty-seven fresh samples of primary cancer and 56 matched non-tumor tissues adjacent to the tumor (NAT) were collected from patients with resectable right-sided colon cancer. NAT were systematically obtained at varying distances (1, 5, and 10 cm) on both proximal and distal sides. Comprehensive gene expression analysis was performed using 770-gene PanCancer Progression Panel, delineating distinctive pathways and functional predictions for each region.

**Results:**

Distinctive gene signatures and pathways exhibited by normal-appearing tissues were discovered at varying distances from cancer. Notably, *SFRP2, PTGDS, COL1A1, IL1B, THBS2, PTGIS, COL1A2, NPR1*, and *BGN* were upregulated, while *ENPEP, MMP1*, and *NRCAM* were downregulated significantly in 1-cm tissue compared to farther distances. Substantial alterations in the extracellular matrix (ECM) and prostaglandin/thromboxane synthesis were significantly evident at the 1-cm distance. Functional analysis indicated enhanced cell viability and survival, alongside reduced cellular death and apoptosis.

**Conclusions:**

Different distances exerted a significant impact on gene alteration within the normal-looking mucosa surrounding primary cancer, influenced by various mechanisms. These findings may highlight potential therapeutic targets related to the ECM and prostaglandin/thromboxane pathways for treatment strategies.

**Supplementary Information:**

The online version contains supplementary material available at 10.1186/s12885-023-11595-7.

## Introduction

Despite the development of numerous innovative interventions, colorectal adenocarcinoma continues to be the most prevalent gastrointestinal malignancy and the second leading cause of cancer mortality worldwide [1, 2]. The recurrence rate following curative treatment, including both distant and locoregional recurrences, for colon cancer is approximately 40%, with local recurrence accounting for 10–20% [3, 4]. The “seed and soil” hypothesis in colorectal cancer refers to the dynamic interplay between cancer cells (seed) and the tumor microenvironment (TME) and immune system (soil) to promote cancer progression at both local and systemic levels. Focusing on cancer cells (seed), surgical removal aims to disrupt this balance by eliminating the cancer cells [5]. Possible etiologies of locoregional recurrences are incomplete removal of tumor cells in bowel wall and mesenteric lymph nodes, tumor spillage, and tumor cell embedment [3, 4]. To ensure complete clearance, surgeons routinely resect primary colon cancer with its grossly normal-looking bowel in both proximal and distal directions, known as the surgical resection margin. However, there is no clear consensus on the optimal resection margin among standard guidelines [6–8]. If the resection margin was greater than 5 cm, the recurrent rate of cancer invaded muscularis propria or deeper without lymph node metastasis decreased from 43 to 9% [9, 10]. Moreover, a surgical margin of 5 cm or greater significantly prolonged the time to recurrence from 21.8 to 32.3 months [11]. Recently, The Japanese Society for Cancer of the Colon and Rectum (JSCCR) found that in patients of node-positive disease, 93.0% of metastatic nodes are found within 0 to 5 cm of the primary tumor, with 7.0% located within the 5 to 10 cm range. This highlights the significance of optimal bowel resection margins in colon cancer surgery [6, 12]. While a longer length of resection in colorectal cancer may achieve a more complete clearance, addressing both horizontal and vertical margins, and potentially improving oncological outcomes, excessive resection length can introduce surgical technical challenges and have adverse effects on patients' quality of life, particularly in terms of bowel habits.

In terms of biomolecular perspective, colonic adenocarcinoma was complicated by numerous genetic and epigenetic alterations, which have led to the identification of several significant therapeutic targets [13–15]. Notably, patients with advanced right-sided colon cancer have a worse prognosis than those with left-sided cancers. Right-sided cancers are associated with higher rates of microsatellite instability (MSI), more frequent aberrant activation of the *EGFR* pathway including higher *BRAF* and *PIK3CA* mutation rates, and increased mutational burden compared to cancers in other locations [16]. Not only interpatient heterogeneity, but temporal heterogeneity and spatial heterogeneity are also significant [17, 18]. On the other hand, the field cancerization theory has been proposed, which posits that changes in non-tumor tissue around cancer either support tumor growth or transform to a precancerous state, albeit mostly in context of squamous cell carcinoma rather than in adenocarcinoma [19, 20]. By histopathology, nuclear atypia could be detectable extended to 5 cm from tumor [21]. However, there is no strong biomolecular evidence to support field cancerization in colonic adenocarcinoma.

The transcriptomic approach, the molecular study based on total RNA complements, is increasingly employed in the field of molecular oncology [22, 23]. One such approach, the Consensus Molecular subtypes (CMS) of colorectal cancer, utilizes RNA sequencing to classify and identify prognostic and predictive factors for systemic treatment [24]. Previously, the main focus has been on the cancer cells (seed), while the impact of the TME and immune system (soil) on the disease has been overlooked. However, TME in colorectal cancer has currently garnered increasing attention in cancer supporting, and it has been observe to exhibit variation among the various CMS subtypes [25]. Although there are few reports on the transcriptomic change of the histopathologically-normal colonic tissue adjacent to the primary tumor, these non-tumor tissues adjacent to tumor (NAT) have their own specific gene expression characteristics that discriminate them from primary cancer and normal tissue from non-diseased donors. This different expression in NAT may be induced by molecular crosstalk from primary tumor through intraluminal bowel environment [26, 27].

Given that different surgical margins can affect the recurrent rate and the patients’ survival, we hypothesize that the varying distances of NAT may also have different genes and pathways. Therefore, this study sought to systematically assess gene activity in the right-sided colon cancer and NAT, and identify potential molecular mechanisms in recurrence and potential therapeutic targets for colon cancers.

## Materials and methods

### Patient cohorts and collection of specimens

**Fig. 1:**
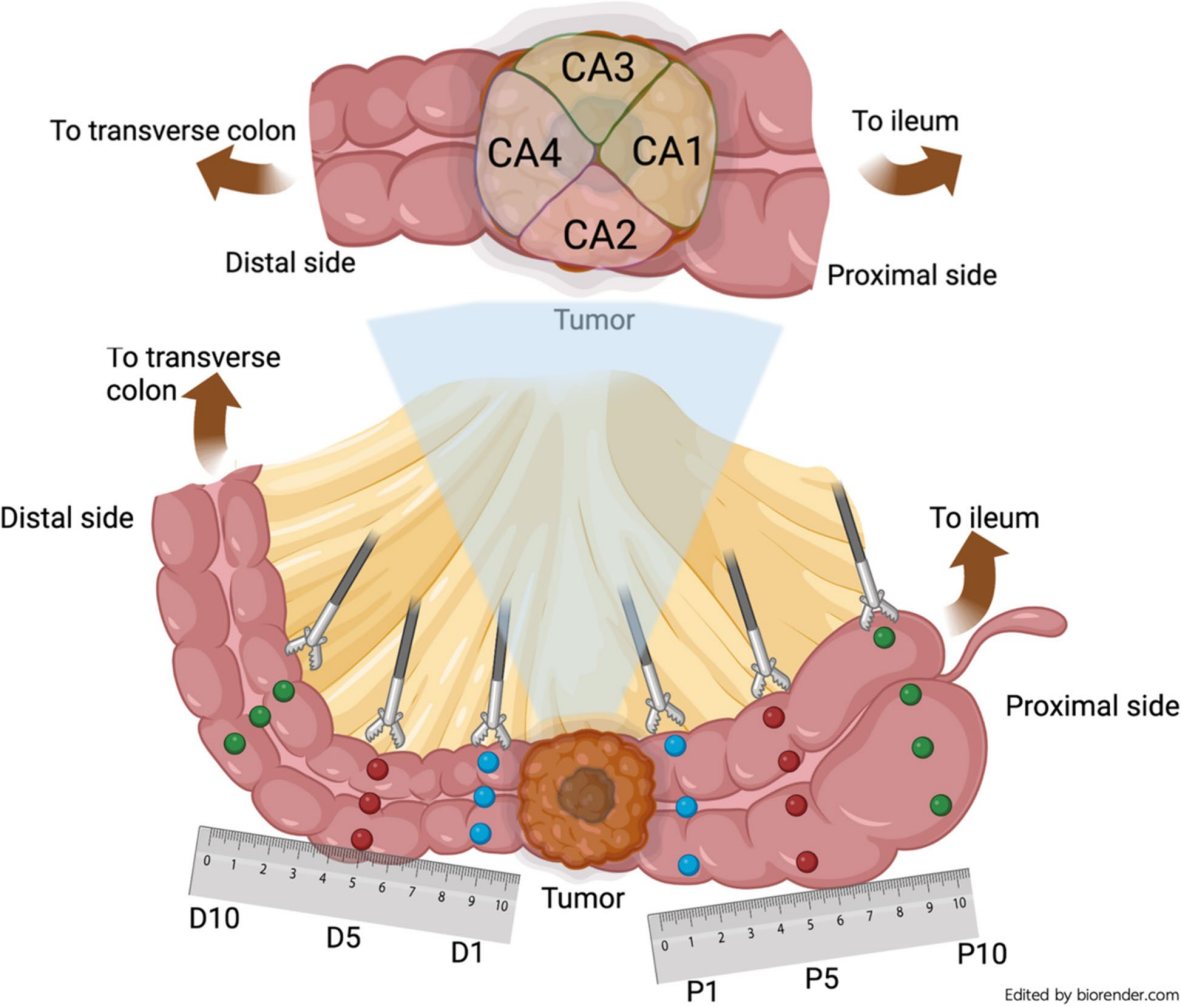
The sampling and coding from fresh surgical specimens of the right-sided colon are presented. NATs located 1, 5, and 10 cm from the gross tumor on the proximal side were represented by P1, P5, and P10, respectively, while NATs located at 1, 5, and 10 cm away from the tumor in distal side were represented by D1, D5, and D10, respectively. CA1, CA2, CA3, and CA4 were cancer samples from the quadrant on the proximal, antimesenteric, mesenteric, and distal sides, respectively. NATs at 1-cm distance (P1 + D1) was defined as peritumoral tissue, while the far-distance tissue was defined as the combination of the 5- and 10-cm normal-appearing specimens (P5 + P10 + D5 + D10)

Patients who underwent curative oncologic surgery for right-sided colonic adenocarcinoma between January 2021 and May 2022 were prospectively recruited. The treatment plan was determined by the attending surgeons according to standard practices. We excluded the patients over the age of 85 years, those with clinical suspicion of distant metastasis, hereditary colorectal cancer, resection margin less than 5 cm, recurrent cases, preoperative systemic therapy or radiation, or tumor smaller than 15 mm. Preoperative bowel preparation was omitted. After the right-sided colon was resected, the researchers immediately collected 2-by-2-mm tissues from both primary cancer and NAT systematically in the operative theatre. Quadrantic sampling was adopted to collect primary cancer samples, randomly selecting 2–3 samples per tumor, in recognition of the potential intratumoral heterogeneity. NATs were taken at 1 cm, 5 cm, and 10 cm from tumor on both proximal and distal sides, excluding the ileum. The sampling was started from the farthest tissue (10 cm) and progressively moving closer (5 cm, 1 cm, and tumor) using separated forceps and scissors to avoid cancer cell contamination. To reduce potential contaminants, all specimens were rigorously cleaned using a separate phosphate-buffered saline (PBS) solution before preservation in 1-mL RNAlater™ stabilization solution (Thermo Fisher Scientific, MA, USA) in a -80 ^O^C freezer [28]. The specimen sampling and coding were presented in Fig. 1.

### RNA extraction and qualification

Fresh specimens weighing 20–30 mg were homogenized using the FastPrep-24™ 5G bead beating grinder and lysis system (MP Biomedicals, CA, USA). The resulting lysate was then subjected to a standard total RNA isolation protocol using RNeasy® Mini Kits (Qiagen, Germany). RNA concentration and purity were measured using Nanodrop™ 8000 spectrophotometer (Thermo Fisher Scientific, MA, USA) and Invitrogen™ Qubit™ 4 fluorometer (Invitrogen™, Thermo Fisher Scientific, MA, USA). RNA purity was assessed by A260/A280 ratio, which was required to be not less than 2.0. The quality of RNA quality was evaluated using RNA integrity number (RIN) by 2100 Bioanalyzer (Agilent Technologies, CA, US), with an acceptable RIN greater than 7.0 [28].

### Multiplex gene expression analysis and CMS classification

Gene expression analysis was conducted using nCounter® PanCancer Progression Panel, which included 770 genes associated with tumorigenesis, progression, and metastasis (nanoString Technologies, WA, USA). A total of 100 ng of RNA from each qualified sample was hybridized with capture probes and reporter probes using CodeSet hybridization at 65^O^C for 18 h. The hybridized specimens were then processed on an automated nCounter® Prep Station, and the resulting nCounter® cartridges were interpreted by the nCounter® Digital Analyzer. The nSolver™ Analysis Software version 4.0 was used to process the data, then raw counts were normalized using 11 housekeeping genes and spiked controls, and using a threshold count value of 20 as background thresholding. The DEGs were determined by ROSALIND software (nanoString Technologies, WA, USA). A threshold of greater than 1.5-fold change (FC) in conjunction with a *p*-value < 0.05 for significant DEG was established. The classical Benjamini–Hochberg method was used to correct *p*-values for multiple t-tests, taking into account the false discovery rate (FDR) bound for the adjusted *p*-value (*p*-Adj) [29, 30]. To categorize CMS subtypes using nanoString platform-based gene expression data, we applied DeepCC, a supervised cancer subtyping model relying on functional spectra. This involved transforming the gene expression dataset into functional spectra linked to biological pathways and employing a trained artificial neural network within the DeepCC (R package version 0.1.1) to classify the data into four subtypes: CMS1, CMS2, CMS3, and CMS4 [25, 31].

### Gene set, pathway, and function analyses

Gene set analysis (GSA) using directed global significant score (DGSS) was analysed based on nanoString annotations v46 to evaluate the overall significance of gene sets that are functionally related. The global significance score of a gene set measured the cumulative evidence for differential expression of genes in a pathway using square root of the mean squared t-statistic of genes. On the other hand, the DGSS determines the tendency to have over- or under-expressed genes, considering the sign of the t-statistics. Gene set, pathway, and function analyses were performed using nSolver™ Data Analysis, ROSALIND™ software, and QIAGEN Ingenuity Pathway Analysis software (IPA 84978992, IPA, Qiagen, Germany) to examine the biological network. Fisher’s exact test was used to calculate *p*-value for enrichment score and Z-score to predict activation/inhibition of molecular function [32, 33].

## Results

### Demographic data

**Fig. 2:**
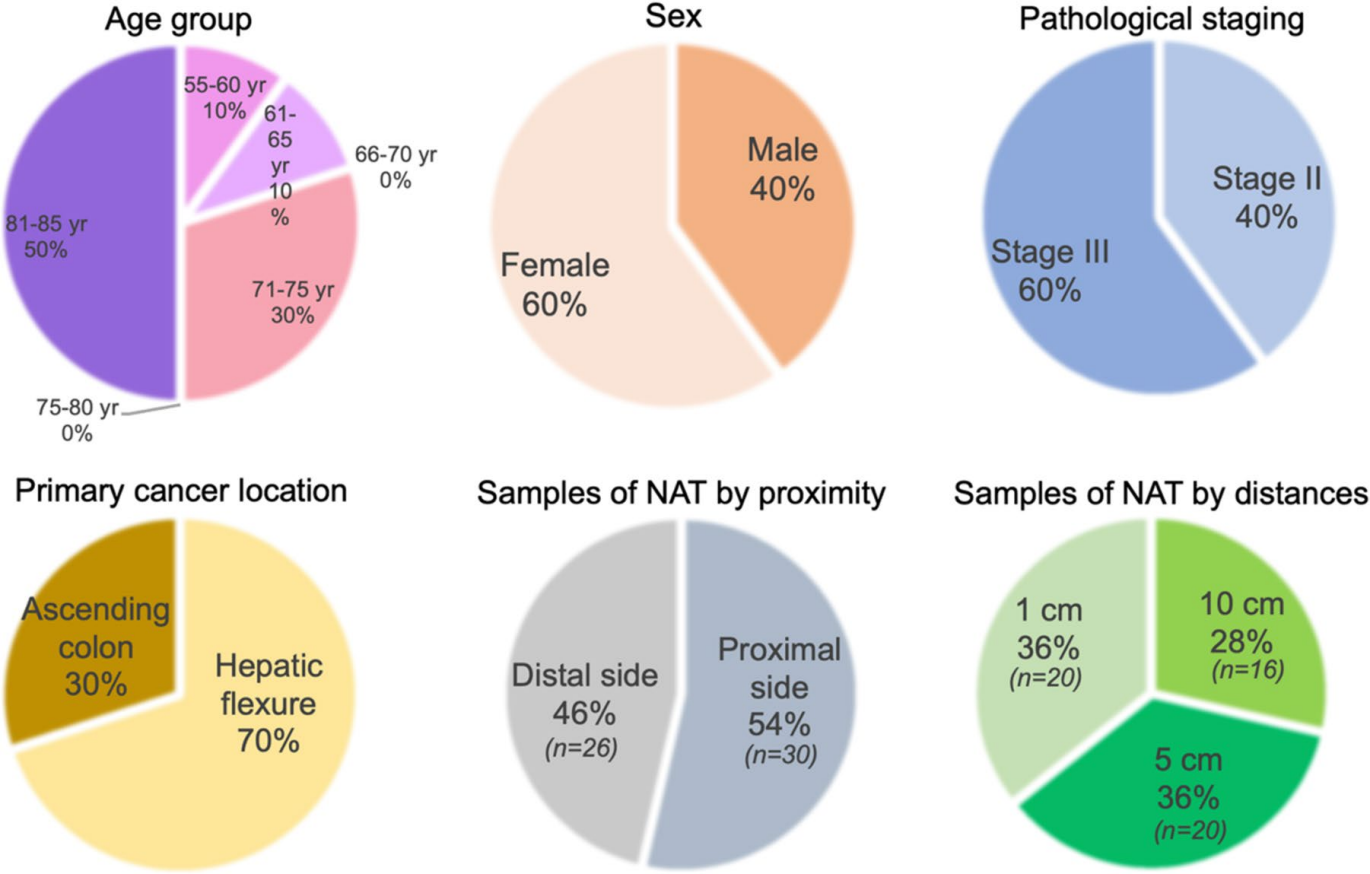
Pie charts illustrate the demographic data of the patients including age, sex, tumor staging, tumor location, and NAT locations

Eighty-three areas were collected from ten patients with right-sided colon cancer, including 27 areas of cancer, 56 areas of matched NAT. The patients consisted of six women and four men with an average age 76.80 ± 9.76 years (mean ± SD). All tumors included were ulceroproliferative lesions, and their boundaries were clearly demarcated. Using deepCC on the cancer specimens, 12 areas (44.44%) were classified as CMS2, two areas (7.41%) as CMS3, 13 areas (48.15%) as CMS4, while no CMS1 was found. Other demographic data including age, staging, differentiation, and locations are also summarized in Fig. 2.

### Gene expressions among surgical resection margins differed from primary cancer

**Fig. 3:**
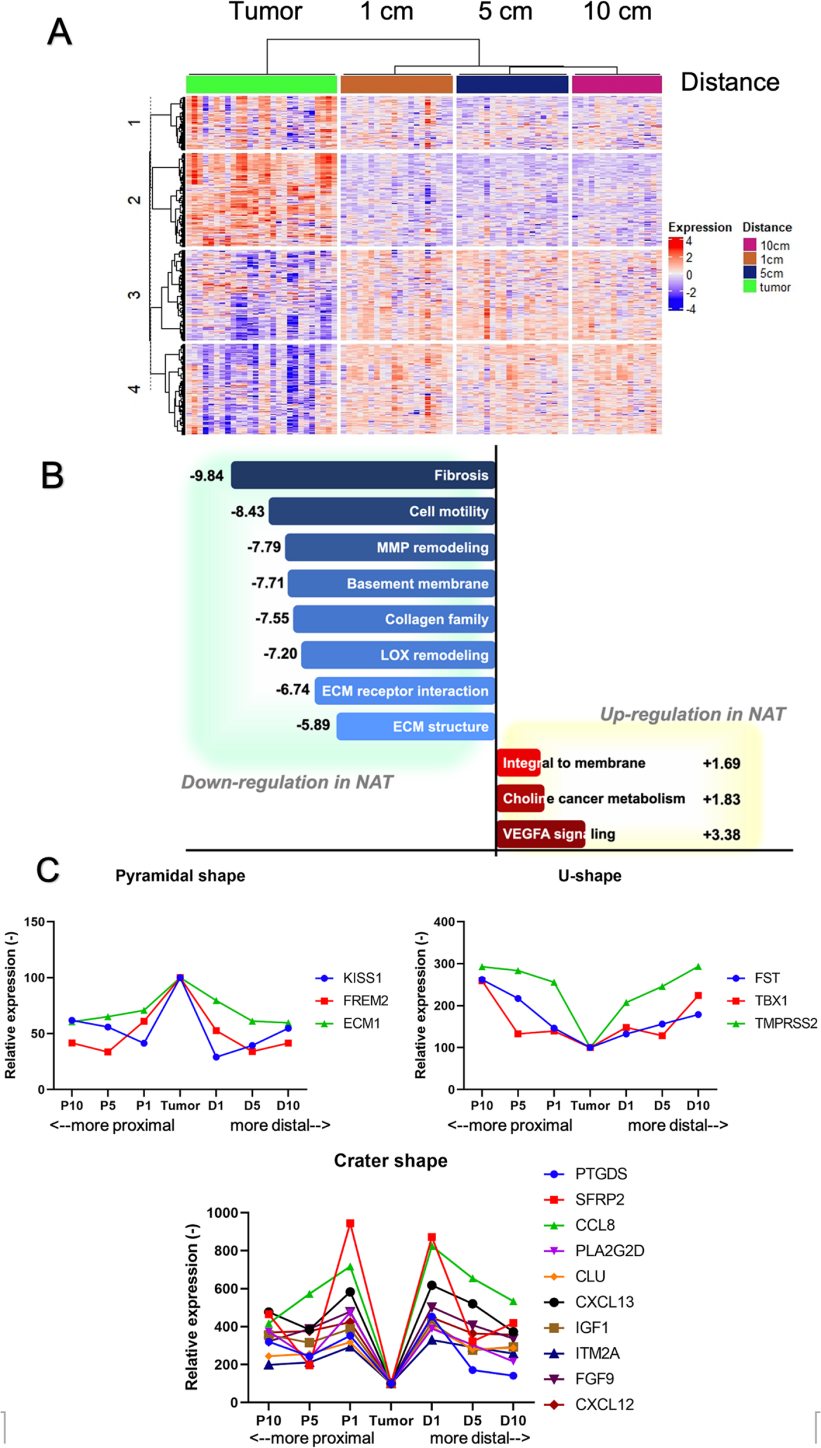
Comparison of expression between primary cancer and NAT by heatmap, GSA, and geographical graphs. **A** shows 316 DEGs between NAT primary and cancer, including 153 upregulated genes and 163 downregulated genes. Expression levels were color-coded, with blue indicating lower levels and red indicating higher levels. Additionally, the column NAT heatmap was also clustered based on the distance from the primary cancer, including the 1-cm, 5-cm, and 10-cm tissues. **B** demonstrates the significantly different gene sets using DGSS between NAT and primary cancer. The figure displays the 8 most downregulated gene sets and 3 upregulated gene sets at NAT, along with their corresponding DGSS values. **C** illustrates the alterations in the relative level of expression to tumor of each DEG as pyramid-shaped, U-shaped, and crater-shaped DEGs using geographical graphs. The Y-axis represented the relative level of expression, while the X-axis indicated sample proximity in the colon. The leftmost and rightmost points corresponded to the most proximal and distal samples, respectively. Each DEG was assigned a different colored line. These patterns suggested that proximity and distance markedly affect the gene expression pattern in NAT

Multiplex gene analysis using nCounter® PanCancer Progression Panel via ROSALIND™ platform, 316 genes showed significant differences between NAT and primary cancer at *p*-Adj 0.05 with an absolute FC greater than 1.5. Among these, 153 genes were upregulated and 163 were downregulated genes in NAT. The heatmap is demonstrated in Fig. 3A.

Next, we performed the GSA based on nanoString annotations v46. The top six upregulated gene sets in primary cancer by DGSS included fibrosis, cell motility, matrix metalloproteinase (MMP) remodeling, basement membrane, collagen family, and lysyl oxidase (LOX) remodeling at DGSS 9.84, 8.43, 7.79, 7.71, 7.55, and 7.20, respectively. Conversely, vascular endothelial growth factor (VEGF) A signaling, choline cancer metabolism, and integral to membrane were upregulated in NAT compared to cancer at DGSS 3.38, 1.83, and 1.69, respectively. Additional gene sets and their corresponding DGSS values are shown in Fig. 3B.

To explore the changes in the expression level related to the proximity of colon and field cancerization, we introduced the parameter ‘relative level of gene expression.’ This parameter measured the ratio of the expression level at a particular point of interest to the cancer level for each DEG. Then, we generated geographical graphs with Y-axis representing the relative gene expression level, and X-axis representing the colon’s proximity, sorted from the most proximal to the most distal. Among these DEGs, we observed three interesting geographical patterns: pyramidal shape, U-shape, and crater shape, along with a non-specific pattern, as shown in Fig. 3C.

Firstly, the pyramidal shape demonstrated maximal upregulation in the tumor and a decrease in gene expression with increasing distance. This pattern included *KISS1*, *FREM2*, and *ECM1*. Secondly, the U-shape pattern depicted the tumor with the lowest level of expression and high expression in the far tissue, including *FST, TBX1*, and *TMPRSS2*. Lastly, a group of genes displayed the highest expression at 1-cm distance and the lowest at the tumor, contributing to the crater shape graph. This gene signature included *PTGDS, SFRP2, CCL8, PLA2G2D, CLU, CXCL12, CXCL13, IGF1, IMT21, and FGF9*. Particularly for *SFRP2*, it was highly expressed at 1-cm distance compared to farther distances.

These findings suggested that NAT differed in gene expression and gene sets compared to primary cancer. The alternating gene expressions observed in geographical patterns between NAT and cancers provide support for the effects of proximity. Gene expression levels in normal-appearing tissues were possibly regulated by primary cancer, with regulation varying based on the distance from cancer in both the proximal and distal regions. This gene expression alteration could be the result of immune response and signaling proteins released from primary cancer or related underlying mechanisms [27, 34].

### NAT from different distances expressed different gene signatures

**Fig. 4:**
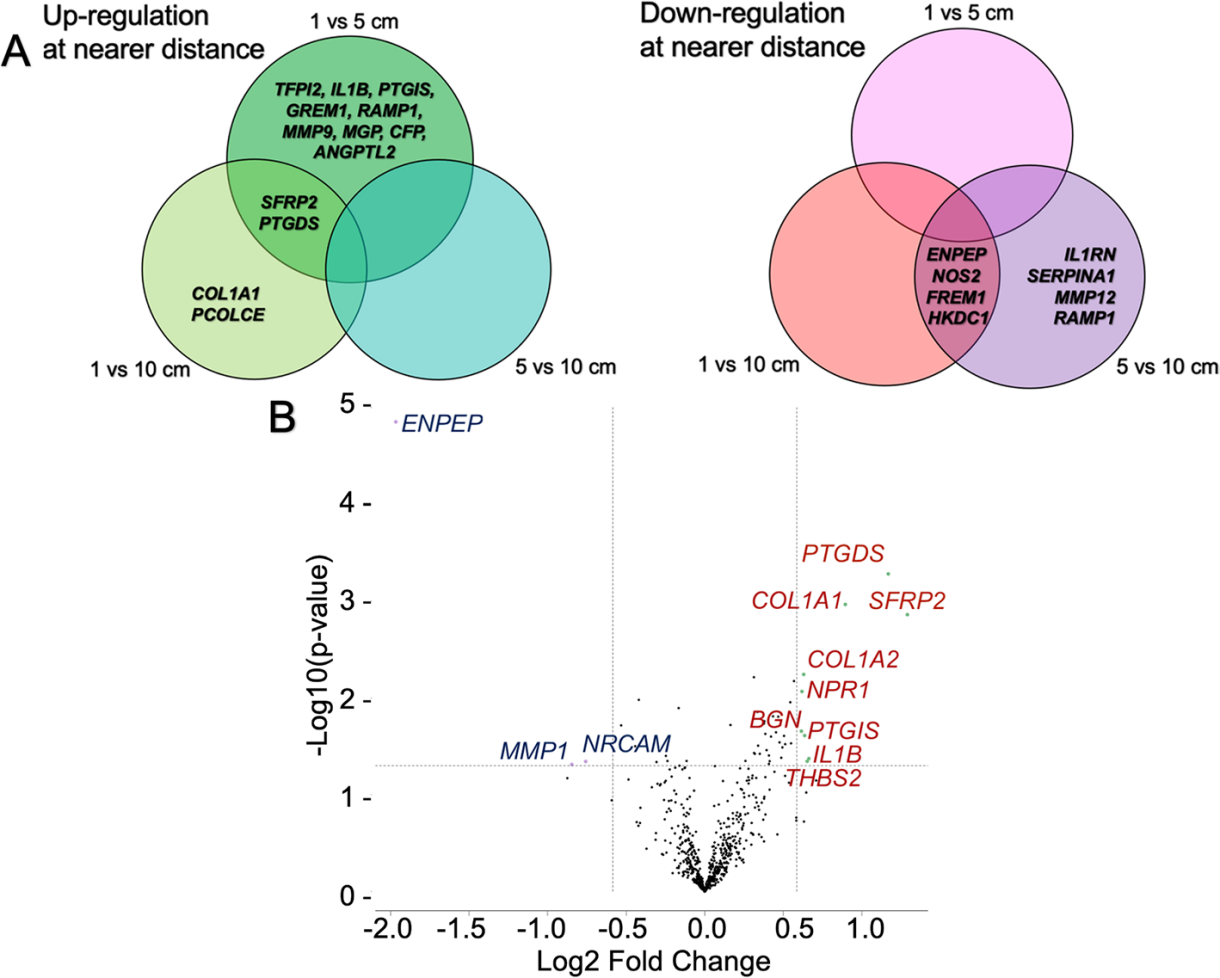
**A** Venn diagrams depict DEGs and common DEGs for each comparison. Among them, *SFRP2* and *PTDGS* were found to be commonly overexpressed in the 1-cm distance group compared to the 5 and 10-cm groups. On the other hand, *ENPEP*, *NOS2*, *FREM1*, and *HKDC1* were commonly overexpressed in the 10-cm distance group compared to the 1- and 5-cm groups. **B** Volcano plot demonstrates DEGs after grouping of P5 + P10 + D5 + D10 samples into “far-distance tissues” category. The X-axis displayed the log_2_ fold change, while the Y-axis represented -log_10_(*p*-value). Among the DEGs, 9 genes were upregulated, while 3 genes were downregulated in the 1-cm samples

To investigate whether the distance from cancer is one of the factors related to intraluminal molecular alteration, we evaluated gene expression levels at distances of 1 cm VS 5 cm, 1 cm VS 10 cm, and 5 cm VS 10 cm. We discovered significant DEGs for each surgical resection margin, with absolute FC greater than 1.5 and *p*-value < 0.05 in both upregulated and downregulated expressions. In addition, we also found the common DEGs for each comparison using Venn diagrams. Among them, *SFRP2* and *PTDGS* were found to be commonly overexpressed at the 1-cm distance compared to the 5 and 10-cm. On the other hand, *ENPEP*, *NOS2*, *FREM1*, and *HKDC1* were commonly overexpressed in the 10-cm distance group compared to the 1 and 5-cm groups. The DEGs and common DEGs were summarized by Venn diagrams in Fig. 4A.

From analysis of DEGs and relative expression levels, the expressions observed at 1-cm distance or peritumoral tissues (P1 + D1) exhibited notable and interesting characteristics. Consequently, we categorized the 5- and 10-cm NAT (P5 + P10 + D5 + D10) as "far-distance tissues." We identified a total of 12 genes that were differentially expressed between 1-cm and far-distance tissues using the criteria of absolute FC greater than 1.5 with *P*-value < 0.05, as shown in Fig. 4B and the supplementary Table S1. The peritumoral tissues exhibited upregulation of nine genes, including *SFRP2, PTGDS, COL1A1, IL1B, THBS2, PTGIS, COL1A2, NPR1*, and *BGN*, while three genes including, *ENPEP*, *MMP1*, and *NRCAM*, were downregulated. The volcano plot of DEGs was illustrated in Fig. 4B.

The findings provided evidence supporting the presence of intraluminal alteration in colon with colon cancer, as evidenced by significant differences in gene expression levels depending on the distance from the tumor. The DEGs identified between peritumoral tissue and far-distance tissues were primarily associated with the regulation of prostaglandins, signaling proteins, and extracellular matrix (ECM).

### Regulation of ECM and prostaglandin-related synthesis play a significant role in the 1-cm area compared to the far-distance tissues

**Fig. 5:**
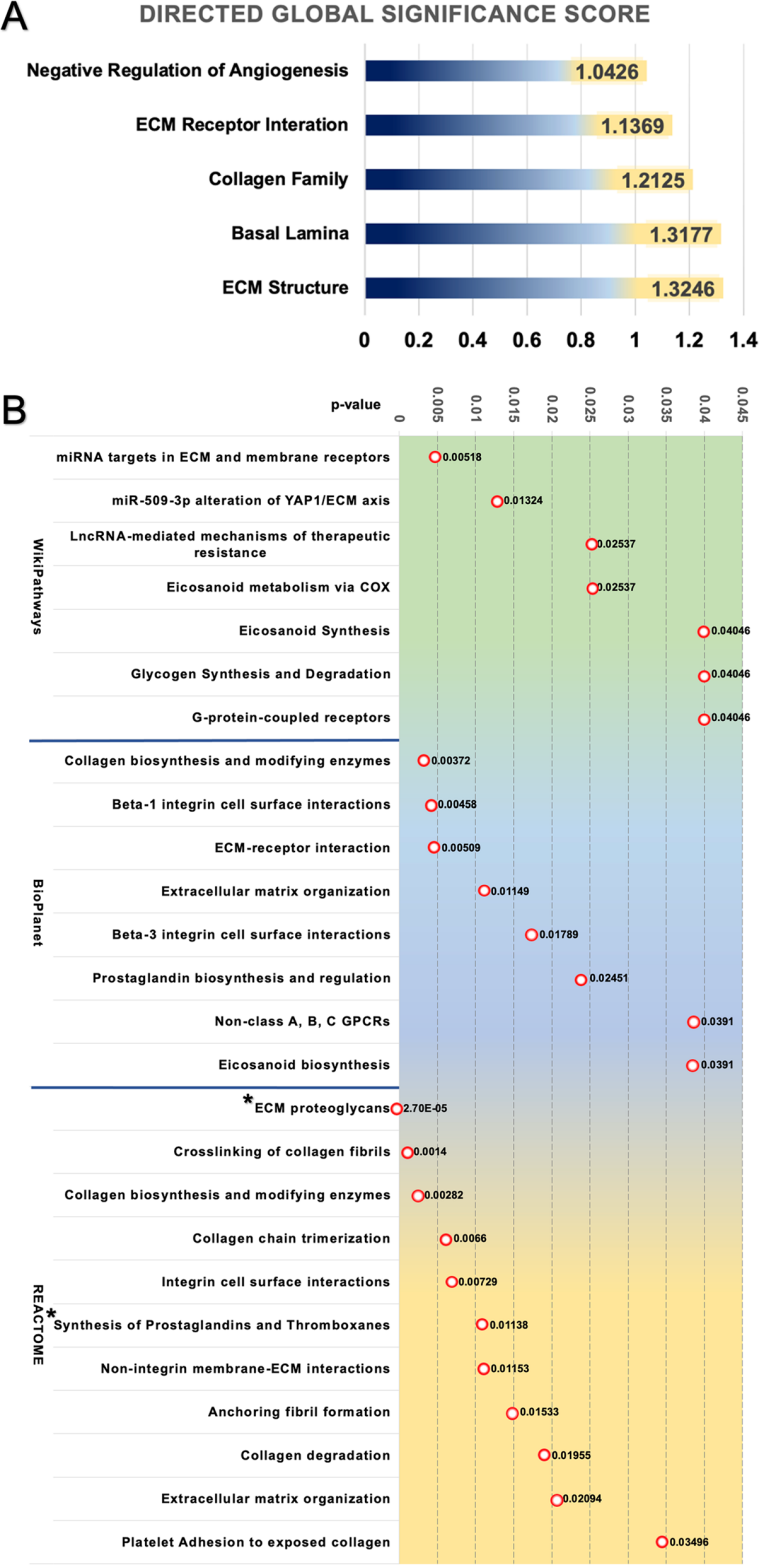
**A** The bar chart displays DGSS of gene sets, which indicates that the functions of ECM structure, basal lamina, collagen family, ECM receptor interaction, and negative regulation of angiogenesis were upregulated in the 1-cm margins compared with far-distance tissues. **B** Significant pathways, identified by WikiPathways, Bioplanet, and REACTOME databases with *p*-value < 0.05 were shown. The *p*-values of each pathway are illustrated in the dotted graphs. Among these pathways, ECM proteoglycan and synthesis of PG/TBX were still significant with *p*-Adj < 0.05, marked by the * sign

We conducted GSA and pathway analysis using 47 DEGs with *p*-value < 0.05. Considering the DGSS of DEGs in 1-cm versus far-distance tissues, the five most highly rated DGSS included ECM structure, basal lamina, collagen family, ECM receptor interaction, and negative regulation of angiogenesis, scoring at DGSS 1.32, 1.32, 1.21, 1.14, and 1.04, respectively, as Fig. 5A. In addition, we further performed pathway analysis using WikiPathways, BioPlanet, PANTHER, and REACTOME databases. Similar to GSA, the pathways related to ECM organization, ECM proteoglycans, miRNA targets in ECM and membrane receptors, ECM-receptor interaction, and Beta-1 integrin cell surface interactions, collagen crosslinking, and collagen biosynthesis and modifying enzymes were significantly altered when compared with far-distance tissue, at a *p*-value < 0.05. Interestingly, we observed significant alterations in pathways associated with eicosanoid and prostaglandin (PG) synthesis in the peritumoral tissues. Furthermore, after correction for multiple testing with FDR (*p*-Adj) < 0.05, ECM proteoglycan and prostaglandin and thromboxane (TBX) synthesis remained significant. (Fig. 5B, details in the supplementary, Table S2).

Our study revealed that the changes in molecular pathways associated with tumor progression were not limited to the primary cancer site, but were also presented in the peritumoral tissues with respect to the distances. These findings suggested ECM and prostaglandin-related pathways play crucial roles in the 1-cm area.

### The biologic functions of cell viability and cell survival are significantly increased at the 1-cm margin

To further investigate the different molecular functions at each resection margin, we conducted a functional analysis using IPA on DEGs with *p*-value < 0.05. Our analysis revealed a total of 20 significant molecular functions related to carcinogenesis and tumor progression, with absolute activation Z-scores greater than 2.0 and *p*-value < 0.05. 17 cellular functions were increased and 3 were decreased in the peritumoral tissue compared to the 5 and 10-cm tissues.

**Fig. 6:**
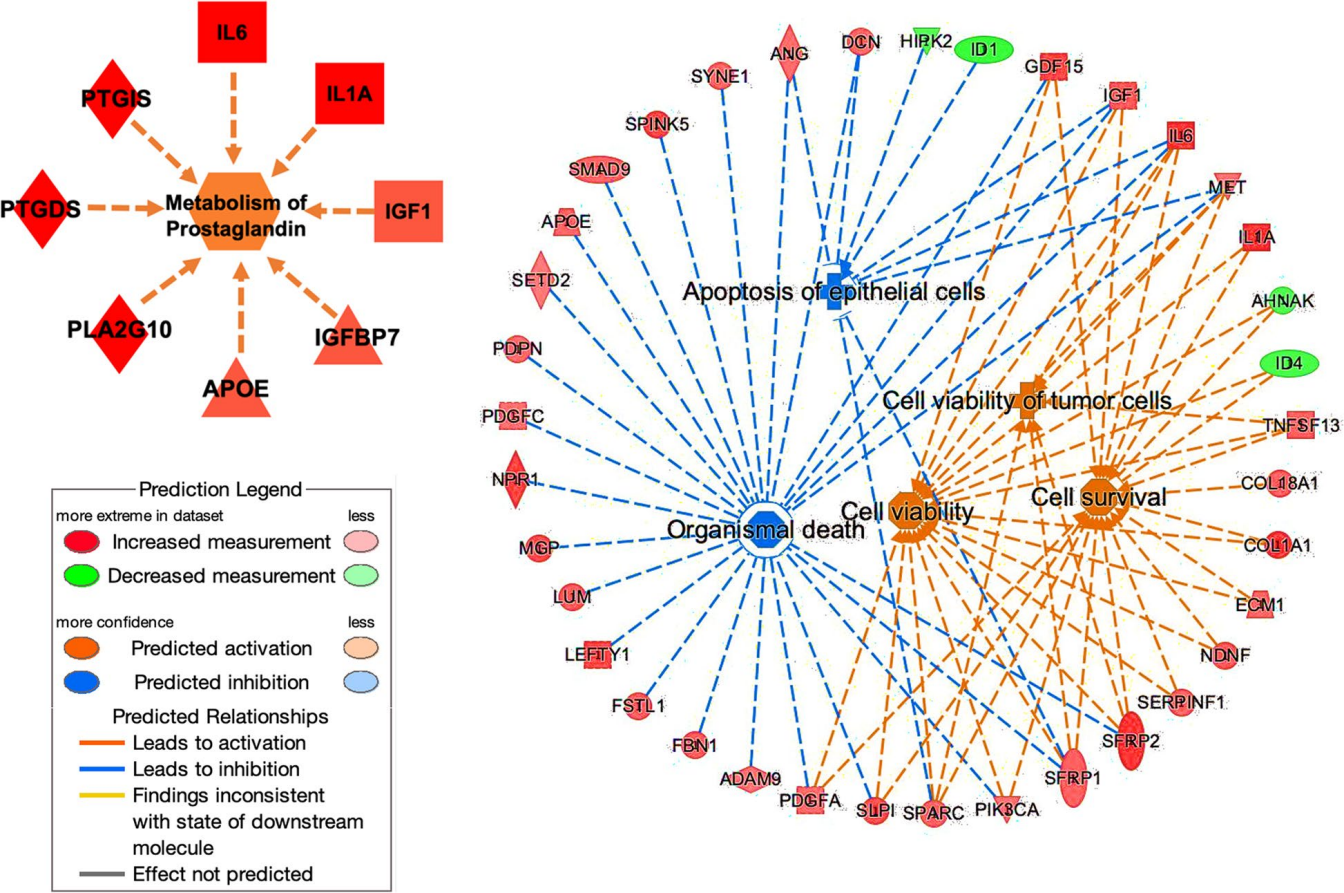
Regulation of DEGs and biological pathways in the 1-cm distance compared to the far-distance tissues using IPA. The prediction legend includes color and intensity to depict the expression levels, inhibitions, activations, and relationships. The gene sets related to metabolism of prostaglandin, cell survival, cell viability, and tumor cell viability were primarily activated, as indicated by the orange arrows. On the other hand, cell death and apoptosis were mainly inhibited, represented by the blue arrow, in the 1-cm tissue

Among these significant molecular functions, prostaglandin metabolism and eicosanoid biosynthesis were increased in 1-cm tissues, consistent with the results of previous pathway analyses. Additionally, the activities of cell viability, cell survival, and tumor cell viability were significantly increased at the 1-cm margin, while organismal death and apoptosis of epithelial cells were decreased compared to the far-distance tissues. The genes related to these findings and their predicted activities are presented in Fig. 6 and the Supplementary Table S3. These observations support the notion that colonic adenocarcinoma not only affected the gene sets but also altered molecular functions among NAT that supported the progression of cancer. The poorer oncological outcomes associated with a narrower surgical resection margin may be due to these underlying molecular functions.

## Discussion

To the best of our current knowledge, the gene expressions among solid tumors, NAT, and healthy colonic mucosa are markedly different [26, 27]. Considering that different surgical resection margins can influence patient survival [9, 10], we hypothesized that the intermediate gene expression properties among NAT vary according to the distance from primary tumor. Furthermore, some early-stage cancers were treated endoscopically with a gross resection margin that is usually smaller than in surgery. Therefore, we conducted this study to emphasize that distance is another crucial factor in molecular expression via gene expression analysis.

While surgery remains crucial for locally advanced colon cancer, less invasive approaches such as endoscopic submucosal dissection (ESD) have emerged as options for early-stage cancer [6–8, 35]. Accordingly, we collected 1-cm, 5-cm, and 10-cm NAT away from primary cancers to represent the biological properties of routine practical surgical margins. In order to minimize the heterogeneity effect [18], we studied only stage 2–3 right-sided cancers without neoadjuvant treatment. We performed an analysis using the PanCancer Progression Panel, which analysis for CMS of colorectal cancer was well validated [36]. The CMS2 and CMS4 subtypes accounted for over 90% of our cancer samples, indicating minimal heterogeneity effect on this study.

First, colonic NAT exhibited deviated genetic regulation from primary colon cancer with 316 DEGs. Interestingly, gene sets related to VEGFA signaling, choline cancer metabolism, integral to membrane, and regulation of angiogenesis were upregulated in NAT, instead of primary cancer. Previous studies showed that NAT expresses unique transcriptomic characteristics that differ from either primary cancer or non-diseased samples. Not only in colorectal cancer, NAT also carried the intermediate state in breast, liver, lung, thyroid, and uterine cancers from the RNA-seq study [26, 37]. Additionally, Hawthorn et al. discovered that the highest enrichment score between primary cancer and NAT was the mismatch repair pathway [34]. Most studies, including our report, have discovered altered gene expressions in NAT of diseased colons. It should be noted that normal-appearing mucosa from patients with colonic lesions is not truly normal and should not be used as the negative control in experiments.

Secondly, DEGs of NAT were influenced by the distance from cancer in the geographical patterns. The concept of ‘field cancerization’ or ‘lateral cancerization’ suggested that primary cancer can induce normal peripheral cells to transform into premalignant and ultimately malignant cells, while also modifying adjacent cellular environments for immortality, progression, and invasion. Recent studies supported this theory by discovering early genetic and/or epigenetic changes without histopathologic abnormality of adjacent cells [20, 38, 39]. Implications of this theory for clinical management are significant, as it suggests that cancer is not just a localized disease, but a process that can affect a larger area of tissue. Taking into account primary cancer, we evaluated the level of expressions of each DEG from each distance concerning cancer, and then finalized in terms of relative level of gene expression and graphical pattern based on the colonic proximity. The relative expression of *KISS1*, *FREM2*, and *ECM1* were in the pyramidal pattern, implying that maximal expression was in the tumor area. In the same way as in previous reports, *FREM2* expression was increased in colorectal cancer foci by immunohistochemistry study and *FREM2* mutation was related to poor oncological outcomes [40]. *ECM1* was oncogenic, which promoted colorectal cancer proliferation and progression via PI3K/AKT/GSK3B/Snail pathway signal [41]. Relative gene expression in the crater shape was interesting due to the maximally expressed level at a distance of 1 cm. The *SFRP* family is the binding antagonist to Wnt signaling, therefore, abnormal methylation of *SFRP* can activate the Wnt pathway, resulting in proliferation and progression of colorectal cancer [42, 43]. Consistent to our report, the gene expression level of *SFRP2* was significantly lowest in the tumor area and highest at a 1-cm distance in both proximal and distal sides, indicating that there was a biomolecular response of NAT to inhibit the tumor lateral growth of primary cancer. *SFRP2* methylation stool testing was recently proposed as a non-invasive colorectal cancer screening tool [44]. These patterns related to altered gene expression may be the result of two possible hypotheses: (1) direct modulation of peritumoral tissues by primary cancer through paracrine signaling or immune response to promote cancer progression, and (2) the response of non-tumor tissue to inhibit cancer proliferation [27, 34].

The alterations of gene sets, pathways, and functions were also influenced by distances. The expression of *ENPEP* was downregulated, while *COL1A1, PTDGS,* and *SFRP2* were upregulated in 1-cm areas compared to 5- and 10-cm areas. *ENPEP* is positively correlated with the aggressiveness of colorectal cancer, and overexpression of *ENPEP* promotes cell migration [45, 46]. In addition to inducing NAT resulting in DEG, primary cancer may also lead to altered ECM, collagen metabolism, and prostaglandin-related pathways based on our pathway analysis. The ECM, which is one of the main components of TME, is composed of various molecules such as collagen, elastin, laminin, fibronectin, and the MMP that play a central role in tissue remodeling and supporting tumor growth [47]. Few previous studies discovered this change in ECM of normal tissues. Tasubo et al. studied the changes in gene expression of peritumoral mesenteric fat in patients with colonic adenocarcinoma, and identified *COL1A1*, *SFRP2*, *FGF7*, *LEF1*, and *CDH1* as DEG when compared to distant fatty tissues. Surprisingly, *COL1A1* and *SFRP2* were upregulated in the nearer area, which was consistent with our intracolonic findings. Additionally, ECM receptor interactions and focal adhesion were upregulated in peritumoral mesenteric fat. This study also suggested the effect of cancer to peritumoral stroma in vertical direction, including mesenteric fat [48]. Both our experiment and previous studies suggested that primary cancers alter the pathways either in vertical and horizontal direction.

In addition to ECM, prostaglandin metabolism was overactivated at 1-cm distance. Previous studies suggest that cyclooxygenase-2 (COX-2) and its proinflammatory metabolite prostaglandin E2 (PGE2) promote colon cancer cell growth through a Gs-axin-beta-catenin signaling axis [49, 50]. Evidence supported the potential utility of non-steroidal anti-inflammatory drugs (NSAIDs) and COX-2 inhibitors in reducing the incidence of adenomatous polyps and chemoprophylaxis of colorectal cancer, suggesting that COX and its products may represent a link between inflammation and colon cancer [50, 51]. Our findings indicated a significant increase in the expression of *PTGDS* and *PTGIS*, which indirectly suggests that the 1-cm area is associated with an increase in prostaglandin metabolites and may be at risk of promoting tumor progression. NSAIDs or COX-2 inhibitors may serve as promising options for adjunct therapy in situations of high risk of recurrence or where resection margin is deemed inadequate.

Lastly, NAT at 1-cm distance was possibly associated with cancer progression and recurrence. Our study identified DEGs that were overexpressed in 1-cm tissues and previously linked to colon cancer recurrence in previous reports. *BGN*, *COL1A1*, and *SFRP2* were upregulated DEGs at 1-cm tissue in our study and these three DEGs were present in the stromal gene group of Connell model related to colon cancer recurrence [52]. Consistently, Zhai et al. discovered genes associated with colon cancer recurrence, which DEGs were also enriched in cellular adhesion and ECM pathways [53]. Our analysis discovered overactivity in pathways of ECM, cell adhesion, collagen-related, and prostaglandin/eicosanoid-related mechanisms. IPA analysis also confirmed the progression-associated function at peritumoral areas, including increase functions of cell viability, cell survival, cell viability of tumor cells and prostaglandin metabolism, while organismal death and epithelial cells apoptosis decreased. However, these functional outcomes may result from mitosis factors that are locally released by the cancers and spread through the neighboring tissue, possibly influenced by elevated VEGF levels. In summary, primary colon cancer that alters peritumoral areas to be associated with cancer progression and recurrence risk.

Limitation of this study was the use of PanCancer Progression Panel which restricted the number to 770 cancer-related genes resulting in low transcriptomic coverage and underpopulation of gene sets. However, this panel has been widely used in numerous cancer studies [36, 54–56]. Moreover, this technique did not require limitations on formalin-fixed paraffin-embedded (FFPE) specimens or RIN, allowing preserved specimens in histopathologic banks to be processed for further study. Nonetheless, as we employed whole fresh tissues and absence of cross-over type sampling, definitive mechanisms regarding of the different expressions remain unclear. Because various factors could contribute to alteration in NAT expression, such as the field cancerization, proliferative effect, pro-tumoral spread related to MMP, reactive processes, immune interactions, microbiome, and the clinical presentation of tumors.

This study had potential clinical applications for colon cancer treatment. Targeting therapeutic molecules in the ECM and prostaglandin pathways may be beneficial for adjuvant treatment in cases of inadequate resection or a high risk of recurrence. Further research on NAT in early colorectal cancer treated by ESD may be beneficial to understand the biologic alteration affected by staging and to identify the appropriate endoscopic resection margin. Integrating RNA sequencing to achieve broader coverage, experiment of NAT cells in vitro or animal models, incorporating cross-over concept, and spatial-omic studies may be useful in elucidating the underlying cancerization mechanism among cell populations. Moreover, studies in rectal cancers may also be valuable, as the length of surgical resection markedly affects ostomy status and quality of life.

## Conclusions

NAT exhibited the distinct gene expressions that were influenced by the distance from primary cancer. These DEGs led to various molecular pathways among different distances. Notably, closer margins were associated with cancer-supporting properties, including ECM pathway, collagen biosynthesis, prostaglandin pathway, higher cellular viability functions, and decreased cell apoptosis. Subsequently, inadequate surgical removal of cancer-supporting tissues may result in poor oncological outcome. It is essential to consider these underlying mechanisms when making clinical decisions regarding surgery in colon cancers.

### Supplementary Information

**Additional file 1:** **Table S1.** Differentially expressed genes (DEG) between the 1-cm and far-distance tissues (5- and 10-cm) with their fold change (FC) and *p*-value, analyzed using the ROSALIND^TM^ platform. **Table S2.** Significant cancer-related pathway alterations with their associated DEGs at 1-cm margins compared with the far-distance tissues. Pathway analysis was experimented by WikiPathways, BioPlanet, and REACTOME databases. ^##^ are the pathways with *p*-Adj < 0.05 after correction of false discovery rate (FDR). **Table S3.** The activation Z-scores of the significantly changed biological functions analyzed by Ingenuity Pathway Analysis (IPA) software in the peritumoral tissues compared to the far-distance tissues.

## Data Availability

The datasets used and/or analysed during the current study are available from the corresponding author on reasonable request.

## Abbreviations

CMS: Consensus Molecular Subtypes of colorectal cancer
COX: Cyclo-oxygenases
DEG: Differentially expressed gene (s)
DGSS: Directed global significance score
ECM: Extracellular matrix
ESD: Endoscopic submucosal dissection
EMT: Epithelial-to-mesenchymal transition
FC: Fold change
FDR: False discovery rate
FFPE: Formalin-fixed paraffin-embedded
GSA: Gene set analysis
lncRNA: Long non-coding ribonucleic acid
LOX: Lysyl oxidase
miRNA: Microribonucleic acid
MMP: Matrix metalloproteinase
MSI: Microsatellite instability
NAT: Non-tumor tissue (s) adjacent to the tumor
NSAID: Non-steroidal anti-inflammatory drug (s)
PBS: Phosphate-buffered saline solution
PG or PTG: Prostaglandin
*p*-Adj: Adjusted *p*-value
RIN: RNA integrity number
RNA: Ribonucleic acid
TBX: Thromboxane
TME: Tumor microenvironment
VEGF: Vascular endothelial growth factor
