# Supplementary material for: Exploring extracellular matrix and prostaglandin pathway alterations across varying resection margin distances of right-sided colonic adenocarcinoma
Source: BMC Cancer. 2023 Dec 7;23:1202. doi: 10.1186/s12885-023-11595-7 (PMC10702019; doi:10.1186/s12885-023-11595-7)
Supplement: Supplementary file 1 — Additional file 1: Table S1. Differentially expressed genes (DEG) between the 1-cm and far-distance tissues (5- and 10-cm) with their fold change (FC) and p-value, analyzed using the ROSALINDTM platform. Table S2. Significant cancer-related pathway alterations with their associated DEGs at 1-cm margins compared with the far-distance tissues. Pathway analysis was experimented by WikiPathways, BioPlanet, and REACTOME databases. ## are the pathways with p-Adj < 0.05 after correction of false discovery rate (FDR). Table S3. The activation Z-scores of the significantly changed biological functions analyzed by Ingenuity Pathway Analysis (IPA) software in the peritumoral tissues compared to the far-distance tissues. [file 12885_2023_11595_MOESM1_ESM.docx]

# Supplementary/Supporting Information

# Table S1 Differentially expressed genes (DEG) between the 1-cm and far-distance tissues (5- and 10-cm) with their fold change (FC) and p-value, analyzed using the ROSALIND^TM^ platform.

| Gene signatures | Fold change | p-value |
| --- | --- | --- |
| Downregulated gene expression in 1-cm compared to far-distance | | |
| *ENPEP* | -3.9077 | < 0.001 |
| *MMP1* | -1.79754 | 0.04535 |
| *NRCAM* | -1.69082 | 0.04535 |
| Upregulated gene expression in 1-cm compared to far-distance | | |
| *BGN* | 1.53159 | 0.02203 |
| *NPR1* | 1.53529 | 0.00855 |
| *COL1A2* | 1.54753 | 0.00569 |
| *PTGIS* | 1.5536 | 0.02443 |
| *THBS2* | 1.57131 | 0.04509 |
| *IL1B* | 1.58301 | 0.04235 |
| *COL1A1* | 1.85921 | 0.00108 |
| *PTDGS* | 2.24659 | < 0.001 |
| *SFRP2^#^* | 2.4443 | 0.00137 |

# Table S2 Significant cancer-related pathway alterations with their associated DEGs at 1-cm margins compared with the far-distance tissues. Pathway analysis was experimented by WikiPathways, BioPlanet, and REACTOME databases. *^##^* are the pathways with p-Adj < 0.05 after correction of false discovery rate (FDR).

|  | Pathways | P-value | Related upregulated genes | Related downregulated genes |
| --- | --- | --- | --- | --- |
| WikiPathways | miRNA targets in ECM and membrane receptors | 0.00518 | *THBS2, COL1A2, TNXB, LAMA4* |  |
|  | miR-509-3p alteration of YAP1/ECM axis | 0.01324 | *COL1A1, THBS2, SPARC* |  |
|  | LncRNA-mediated mechanisms of therapeutic resistance | 0.02537 | *MEG3* | *CDKN1* |
|  | Eicosanoid metabolism via Cyclo Oxygenases (COX) | 0.02537 | *PTGDS, PTGIS* |  |
|  | Eicosanoid Synthesis | 0.04046 | *PTGDS, PTGIS* |  |
|  | Glycogen Synthesis and Degradation | 0.04046 |  | *HKDC1, PPP2R1A* |
|  | G-protein-coupled receptors (GPCRs), Other | 0.04046 | *SSTR2, ADGRG1* |  |
| BioPlanet | Collagen biosynthesis and modifying enzymes | 0.00372 | *COL1A1, COL1A2, PCOLCE, COL18A1, COL4A2* |  |
|  | Beta-1 integrin cell surface interactions | 0.00458 | *COL1A1, THBS2, COL1A2, FBN1, ITGA7, LAMA4, COL18A1* | *ITGA8* |
|  | ECM-receptor interaction | 0.00509 | *COL1A1, THBS2, COL1A2, TNXB, ITGA7, LAMA4, AGRN, COL4A2* | *ITGA8* |
|  | ECM organization | 0.01149 | *COL1A1, COL1A2, PCOLCE, COL18A1, COL4A2* | *MMP1* |
|  | Beta-3 integrin cell surface interactions | 0.01789 | *COL1A1, COL1A2, THY1, FBN1, LAMA4* |  |
|  | Pathways | P-value | Related upregulated genes | Related downregulated genes |
| BioPlanet (cont’) | Prostaglandin biosynthesis and regulation | 0.02451 | *PTGDS, PTGIS* |  |
|  | Non-class A, B, C GPCRs | 0.03910 | *SSTR2, ADGRG1* |  |
|  | Eicosanoid biosynthesis | 0.03910 | *PTGDS, PTGIS* |  |
| REACTOME | ECM proteoglycans*^##^* | 2.7^e-05^(p-Adj. 0.00405) | *COL1A1, COL1A2, BGN, TNXB, ITGA7, LAMA4, AGRN, FMOD, SPARC, COL4A2* | *ITGA8* |
|  | Crosslinking of collagen fibrils | 0.00140 | *COL1A1, COL1A2, PCOLCE, COL4A2* |  |
|  | Collagen biosynthesis and modifying enzymes | 0.00282 | *COL1A1, COL1A2, PCOLCE, COL18A1, COL4A2* |  |
|  | Collagen chain trimerization | 0.00660 | *COL1A1, COL1A2, COL18A1, COL4A2* |  |
|  | Integrin cell surface interactions | 0.00729 | *COL1A1, COL1A2, FBN1, ITGA7, AGRN, COL18A1, COL4A2* | *ITGA8* |
|  | Synthesis of Prostaglandins (PG) and Thromboxanes (TBX) *^##^* | 0.01138 (p-Adj. 0.03253) | *PTGDS, PTGIS* |  |
|  | Non-integrin membrane-ECM interactions | 0.01153 | *COL1A1, COL1A2, LAMA4, AGRN, COL4A2* |  |
|  | Anchoring fibril formation | 0.01533 | *COL1A1, COL1A2, COL4A2* |  |
|  | Collagen degradation | 0.01955 | *COL1A1, COL1A2, COL18A1, COL4A2* | *MMP1* |
|  | ECM organization | 0.02094 | *COL1A1, COL1A2, COL4A2* |  |
|  | Platelet Adhesion to exposed collagen | 0.03496 | *COL1A1, COL1A2* |  |

Table S3 The activation Z-scores of the significantly changed biological functions analyzed by Ingenuity Pathway Analysis (IPA) software in the peritumoral tissues compared to the far-distance tissues

| Increased function at 1-cm tissue | | | Decreased function at 1-cm tissue | | |
| --- | --- | --- | --- | --- | --- |
| Functions | Activation Z-score | p-value | Functions | Activation Z-score | p-value |
| Expression of RNA | 2.28 | < 0.01 | Organismal death | -2.73 | < 0.01 |
| Organization of cytoskeleton | 2.02 | < 0.01 | Apoptosis of epithelial cells | -2.26 | < 0.01 |
| Metabolism of prostaglandin | 2.58 | < 0.01 | Damage of vascular system | -2.04 | < 0.01 |
| Cancer of secretory structure | 2.43 | < 0.01 |  |  |  |
| Transcription | 2.09 | < 0.01 |  |  |  |
| Cell viability of tumor cells | 2.43 | < 0.01 |  |  |  |
| MAPKKK cascade | 2.14 | < 0.01 |  |  |  |
| Activation of cell-to-cell signaling | 2.55 | < 0.01 |  |  |  |
| Cell spreading | 2.19 | < 0.01 |  |  |  |
| Lymphocyte migration | 2.34 | < 0.01 |  |  |  |
| Protein kinase cascade | 2.16 | < 0.01 |  |  |  |
| Cell viability | 2.52 | < 0.01 |  |  |  |
| Synthesis of nitric oxide | 2.17 | < 0.01 |  |  |  |
| Cell survival | 2.63 | < 0.01 |  |  |  |
| Migration of mononuclear leukocyte | 2.56 | < 0.01 |  |  |  |
| Cell movement of epithelial cells | 2.25 | < 0.01 |  |  |  |
| Fibrogenesis | 2.06 | < 0.01 |  |  |  |
